# Slowdown of the Walker circulation at solar cycle maximum

**DOI:** 10.1073/pnas.1815060116

**Published:** 2019-03-29

**Authors:** Stergios Misios, Lesley J. Gray, Mads F. Knudsen, Christoffer Karoff, Hauke Schmidt, Joanna D. Haigh

**Affiliations:** ^a^Department of Physics, Oxford University, Oxford, OX1 3PU, United Kingdom;; ^b^Department of Geoscience, Aarhus University, DK-8000 Aarhus C, Denmark;; ^c^National Centre for Atmospheric Science, Oxford, OX1 3PU, United Kingdom;; ^d^iCLIMATE Interdisciplinary Centre for Climate Change, Aarhus University, DK-8000 Aarhus C, Denmark;; ^e^Stellar Astrophysics Centre, Department of Physics and Astronomy, Aarhus University, DK-8000 Aarhus C, Denmark;; ^f^Max Planck Institute for Meteorology, 20146, Hamburg, Germany;; ^g^Department of Physics, Imperial College London, London, SW7 2BW, United Kingdom;; ^h^Grantham Institute, Imperial College London, London, SW7 2BW, United Kingdom

**Keywords:** 11-y solar cycle, Walker circulation, GHG forcing, climate model

## Abstract

The Pacific Walker Circulation (PWC) fluctuates on interannual and multidecadal timescales under the influence of internal variability and external forcings. Here, we provide observational evidence that the 11-y solar cycle (SC) affects the PWC on decadal timescales. We observe a robust reduction of east–west sea-level pressure gradients over the Indo-Pacific Ocean during solar maxima and the following 1–2 y. This reduction is associated with westerly wind anomalies at the surface and throughout the equatorial troposphere in the western/central Pacific paired with an eastward shift of convective precipitation that brings more rainfall to the central Pacific. We show that this is initiated by a thermodynamical response of the global hydrological cycle to surface warming, further amplified by atmosphere–ocean coupling, leading to larger positive ocean temperature anomalies in the equatorial Pacific than expected from simple radiative forcing considerations. The observed solar modulation of the PWC is supported by a set of coupled ocean–atmosphere climate model simulations forced only by SC irradiance variations. We highlight the importance of a muted hydrology mechanism that acts to weaken the PWC. Demonstration of this mechanism acting on the 11-y SC timescale adds confidence in model predictions that the same mechanism also weakens the PWC under increasing greenhouse gas forcing.

## Significance

Influences of the 11-y solar cycle (SC) on climate have been speculated, but here we provide robust evidence that the SC affects decadal variability in the tropical Pacific. By analyzing independent observations, we demonstrate a slowdown of the Pacific Walker Circulation (PWC) at SC maximum. We find a muted hydrological cycle at solar maximum that weakens the PWC and this is amplified by a Bjerknes feedback. Given that a similar muted hydrological cycle has been simulated under increased greenhouse gas forcing, our results strengthen confidence in model predictions of a weakened PWC in a warmer climate. The results also suggest that SC forcing is a source of skill for decadal predictions in the Indo-Pacific region.

The Pacific Walker Circulation (PWC) consists of a large-scale zonal overturning atmospheric circulation pattern over the equatorial Indian and Pacific Oceans that plays a key role in global climate by redistributing heat and precipitation. On interannual timescales, the PWC is tightly coupled to local variations of sea-surface temperatures (SSTs) associated with the El Niño Southern Oscillation (ENSO); its warm phase causes reduced sea-level pressure (SLP) gradients between the western and eastern Pacific, weaker trade winds, an eastward displacement of the PWC, and increased rainfall in the central/east Pacific (1). Model simulations of climate change indicate an overall weakening of the PWC, explained in terms of a muted hydrology cycle as global-mean precipitation increases (2–3%/K of surface warming) at a lower rate than atmospheric humidity (∼7%/K following the Clausius–Clapeyron, C-C, relationship) (2, 3). However, observational evidence of PWC trends in the 20th century is contradictory, depending on the diagnostics examined and which time period is selected, especially as the last few decades have been characterized by a number of La Niña events that result in a strengthened rather than a weakened PWC (4–6).

In view of the inconclusive observational evidence for the greenhouse gas (GHG) response, one approach is to look for improved insight by examining the transient response of tropical climate to other external factors. Examination of the response to the 11-y solar cycle (SC) forcing presents such an opportunity. There is a variety of observational evidence for an SC influence at the Earth’s surface, generally interpreted in terms of a “top-down” stratospheric influence via UV irradiance and ozone changes and “bottom-up” influences via total solar irradiance (TSI) and hence surface heating (7). The bottom-up mechanism is most likely the primary influence in the Indo-Pacific (7–9), but there have been contradictory interpretations of the observations. Composite analyses have associated SC maximum (Smax) years with negative SST anomalies in the equatorial Pacific, stronger east–west SLP gradient, enhanced surface easterlies, and ultimately a stronger PWC (10–12). However, ENSO variability may severely contaminate signals in composites of limited time periods (13, 14). Alternative approaches such as multiple regression analysis that take into account variability associated with ENSO and other forcings (e.g., volcanic) suggest a warmer equatorial Pacific Ocean (∼0.4 K) lagging Smax by 1–2 y (13, 15), which slackens the east–west SLP gradient and reduces the strength of the PWC. Given that the radiative imbalance at the top of the atmosphere barely exceeds 0.18 W/m^2^ between Smin and Smax, radiative forcing alone suggests a very modest global-mean surface warming of only 0.08–0.16 K (15). The considerably stronger sensitivity detected in the Pacific is thought to be due to feedbacks between the direct surface heating, clouds/precipitation changes, and dynamical ocean adjustments (7–9).

A weakened PWC in response to Smax has been identified, albeit with varying strength, in the majority of climate models. Responding to warmer Pacific SSTs at Smax, the models simulate an increase in tropical precipitation (16), deep convection shifts from the maritime continent toward the central Pacific, and the easterly trade winds slacken, the latter indicating a weakening and eastward displacement of the upward branch of the PWC (17). Extensive examination of the historical simulations in the fifth phase of the Coupled Model Intercomparison Project (CMIP5) (18) attributed the weakened PWC response to the weaker sensitivity of global precipitation to surface warming (∼1.9%/K) compared with global atmospheric moistening (∼6.6%/K).

It turns out, therefore, that the muted hydrological cycle mechanism may slow down the PWC under both SC and GHG forcings, at least in climate models. Here, we demonstrate the transient response of the PWC to the SC in a variety of independent observational records spanning the second half of the 20th century. The periodic nature of SC forcing reduces the potential influence of uncorrected observational biases in existing datasets that often hamper the detection of longer-term trends. Our methodology is based on a simple lead/lag multiple-linear regression (MLR) that includes ENSO, volcanic, and GHG forcings, as well as the SC (*Materials and Methods*). We find a clear, statistically significant weakening of the PWC at Smax. Idealized model simulations support the observational pattern of a weakened PWC, and are interpreted in terms of the bottom-up mechanism, initiated by a zonally uniform SST warming and amplified by an ocean–atmosphere (Bjerknes) feedback.

## Weakening of the Walker Circulation

**Fig. 1.**
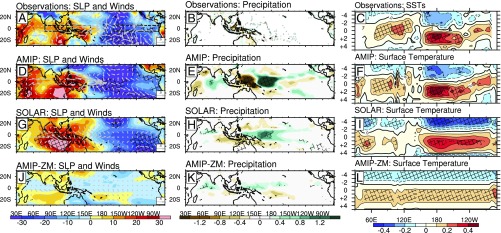
(*Top Row*) SC regression coefficients of (*A*) SLP with 10-m surface winds imposed as arrows, (*B*) precipitation, and (*C*) SSTs averaged over 5°S–5°N. The SLP surface winds and precipitation patterns are shown at +1 y time lag; SC signals in SSTs are shown as a Hovmøller plot of lead/lagged (±5 y, positive lag indicates a response that lags the SC). Units are Pa, ms^−1^, mm/d, and K per 1-W/m^2^ increase in TSI, respectively. SLP data from HadSLP2r, surface wind data from WASwind, precipitation data from GPCC, SSTs from Kaplan, all datasets from 1950 onward. Hatched areas indicate chance probability *P* < 0.1. Following rows indicate the corresponding signals from the ensemble average of the AMIP (*D*–*F*), SOLAR(*G*–*I*), and AMIP-ZM (*J*–*L*) simulations.

We first isolate spatially coherent patterns of annual-averaged tropical (30°S–30°N) SLP anomalies in the Indian and Pacific basins. Although the HadSLP2r dataset (*Materials and Methods*) spans the whole 20th century, we focus on the second half of the 20th century (1950–2013), for improved data quality, at the expense of a reduced sample length. The spatial pattern of the solar SLP regression coefficients (Fig. 1*A*) at lag +1 y has positive anomalies over the maritime continent and negative anomalies in the central and eastern Pacific, the latter demonstrating a chance probability of *P* < 0.1 according to the Cochrane–Orcutt method. This pattern, which indicates an overall relaxation of the climatological zonal SLP gradient, remains robust even over the whole period 1900–2013 or under different configurations of the MLR model (*SI Appendix*, Fig. S1) and is present in individual seasons (13). The ΔSLP difference between the equatorial (5°S–5°N) Indian/West Pacific (160°–80°W) and East Pacific (80°–160°E) (boxes in Fig. 1*A*), which serves as a proxy of the PWC strength (5), has a positive loading (24–28 Pa for a typical SC) both over 1900–2013 (*P* < 0.1) and 1950–2013 (*P* < 0.05) (*SI Appendix*, Fig. S2). This means that the climatological ΔSLP gradient slackens by about 13% from Smin to Smax, a response about an order of magnitude weaker than the reduction during typical El Niño events.

**Fig. 2.**
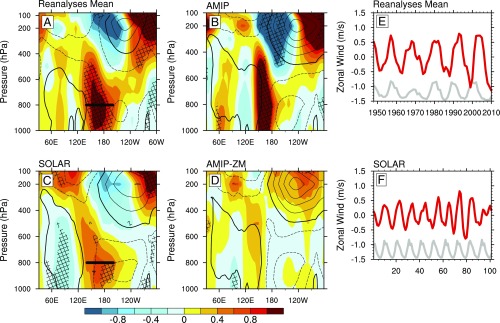
SC regression coefficients of equatorial zonal winds for (*A*) the multireanalysis mean of 20CR, ERA20, and NCEP, (*B*) AMIP ensemble mean, (*C*) SOLAR ensemble mean, and (*D*) AMIP-ZM ensemble mean. Hatched areas indicate chance significance *P* < 0.1. Contour lines show the zonal wind climatology. Units in ms^−1^ per 1-W/m^2^ increase in TSI. Signals refer to +1-y time lag. (*E* and *F*) MSSA filtered equatorial zonal winds (ms^−1^) in the multireanalysis mean composite (1950–2010) and SOLAR, respectively, averaged over 800 hPa and 140°E–160°W (marked in *A* and *C*). TSI variability (gray, normalized) is superimposed for reference.

Positive ΔSLP anomalies imply weakened surface easterlies and this is evident in the wave- and anemometer-based sea surface wind (WASWind) surface wind observations from 1950 to 2011 (*Materials and Methods*), which show westerly wind anomalies of 0.7–1.2 ms^−1^ over the western and central Pacific (arrows in Fig. 1*A*), with a Pacific-basin–wide (120°E–70°W, 5°S–5°N) mean reduction of ∼0.1 ms^−1^ (*P* < 0.3). To study the vertical structure of the wind anomalies and in the absence of dense direct observations, we analyze equatorial zonal winds in a multireanalysis composite [mean of NOAA-CIRES 20th century (20CR), ERA 20th century (ERA20), NCEP/NCAR reanalysis (NCEP)] over the common period 1950–2010, but we note that similar signatures are found over the full 20th century (*SI Appendix*, Fig. S3). Despite some differences among the reanalyses, a statistically significant reduction of climatological equatorial easterlies is evident in the central Pacific, penetrating high into the troposphere up to ∼400 hPa, with anomalies exceeding 1 ms^−1^ at 800 hPa (*P* < 0.05, Fig. 2*A*). The dipole of positive/negative wind anomalies straddling the climatological westerlies in the upper troposphere (e.g., 200 hPa, 180°–60°W) indicates an eastward displacement of the ascending branch of the PWC toward the central Pacific.

**Fig. 3.**
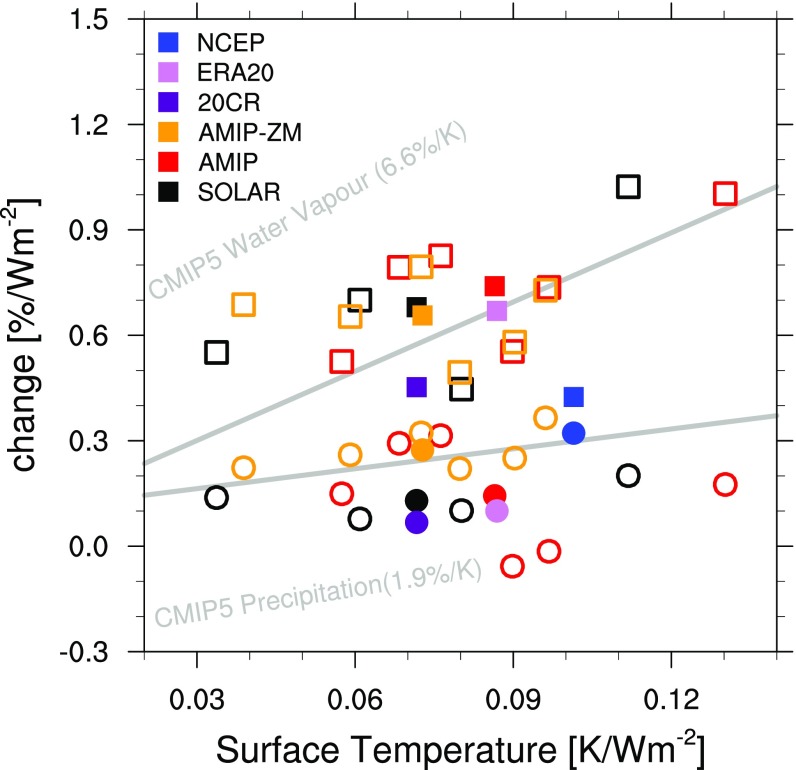
Comparison of SC regression coefficients (percentage changes) of global-mean precipitation (circles) and WVC (squares) against the respective global-mean surface temperature change. Units in percent and K per 1-W/m^2^ increase in TSI, respectively. Open circles and squares mark individual ensemble members of the SOLAR, AMIP, and AMIP-ZM simulations. All signals refer to +1-y time lag. Gray lines indicate sensitivities to SC inferred in the CMIP5 20th century simulations (18).

To validate these MLR results, we analyze time series of zonal wind anomalies averaged over the equatorial western and central Pacific troposphere (800 hPa, 140°E–160°W, black line in Fig. 3*A*) using a multichannel singular spectrum analysis approach (MSSA) that identifies quasiperiodic oscillations (19). The MSSA (nine channels with 15-y window) isolates an 11.9-y quasiperiodic oscillation that tracks TSI variations throughout the analyzed period with a correlation coefficient of 0.74 peaking at 1–2-y lag (Fig. 2*E*). The amplitude of the detected decadal oscillation in this region exceeds 1 ms^−1^, consistent with the MLR signal, with prevalent westerly anomalies in Smax reversing to easterly anomalies during Smin years.

A weaker and eastward-shifted PWC at Smax results in an eastward migration of atmospheric convection and precipitation maxima toward the central Pacific as evident in the Global Precipitation Climatology Centre (GPCC) precipitation dataset (*Materials and Methods*) over the period 1950–2013 (Fig. 1*B*). Despite the limited spatial coverage of GPCC in the Pacific, the detected pattern is robust and is supported by the reanalyses and the independent GPCP v.2.3 global precipitation dataset (*SI Appendix*, Fig. S4).

The observed signals in Figs. 1 and 2 refer to 1 y after Smax (lag +1 y), when the strongest ΔSLP anomalies are detected in HadSLP2r and the reanalyses (*SI Appendix*, Fig. S5). This time lag is similar to that identified in previous studies (15, 18) and indeed our MLR analysis of the Kaplan SSTs over the period 1950–2013 (*Materials and Methods*) detects a significant SST warming of more than 0.4 K over the equatorial Pacific that maximizes at 1-y time lag (Fig. 1*C*). The spatial pattern of these SST anomalies is characterized by a stronger loading over the central Pacific and overall warming over the Indian Ocean, as shown in earlier MLR analyses (13, 18, 20).

In line with other studies, we propose that the observed SLP, wind, and precipitation anomalies are all associated with this SST warming. To highlight the bottom-up contribution of SSTs, we perform a six-member ensemble of an atmosphere-only global climate model for 1950–2013 (33 SCs) forced only at the surface boundary by time-varying SST and sea-ice concentration observations (AMIP ensemble, *Materials and Methods*), while deliberately keeping the solar irradiance and all other forcings constant. The SLP regression coefficients show good agreement with the HadSLP2r at lag +1 y, and significant positive/negative SLP anomalies are simulated over the Indian Ocean/West Pacific and the East Pacific, respectively, resulting in a positive ensemble mean ΔSLP of +41 Pa at Smax (Fig. 1*D* and *SI Appendix*, Figs. S2 and S5). Westerly surface wind anomalies (>1 ms^−1^) are simulated between 150° and 170°W that extend up to 400 hPa into the equatorial troposphere in all individual AMIP runs, although the ensemble mean westerly anomalies are narrower than in reanalyses with an overall stronger magnitude (1.5 ms^−1^ at 800 hPa), likely explained by differences in the extent and position of the climatological PWC (Fig. 2*B* and *SI Appendix*, Fig. S6). Consistent with precipitation observations, the upward branch of the PWC shifts eastward bringing significantly more rainfall (>2.5 mm d^−1^) toward the central Pacific (Fig. 1*E*).

While the AMIP ensemble underlines the role of SST anomalies, this is insufficient evidence for the role of SC forcing on the PWC. Although the null hypothesis, that the observed responses simply reflect internally generated decadal variability, can be rejected in statistical terms with probability <0.1, the MLR analysis alone cannot establish causal linkages between SC forcing and warmer SSTs/weakened PWC per se. In fact, the detected patterns in tropical PWC, SSTs, and precipitation share common features with modes associated with internal Pacific variability (21), implying a possible signal contamination. To demonstrate a physical linkage to SC forcing, we perform a four-member, 100-y ensemble (∼40 SCs) using a coupled atmosphere–ocean model forced only by repeating the solar cycle 22 (1986–1996) variation in solar spectral irradiance (SOLAR ensemble, *Materials and Methods*).

Increased TSI at Smax in the SOLAR results in a widespread warming over the equatorial Pacific, with regression coefficients of up to +0.4 K that maximize at a lag of 1–2 y, in good agreement with the observations (Fig. 1*I*). The equatorial SLP responds to the positive SST anomalies by increasing over the Indian Ocean/West Pacific and reducing in the East Pacific and the strongest SLP signal is also found at 1–2 y lag (Fig. 1*G* and *SI Appendix*, Fig. S5). The ensemble mean ΔSLP change of +38 Pa per 1 W/m^2^ increase in TSI is consistent with the observed change of about +24 Pa per 1 W/m^2^ (*SI Appendix*, Fig. S2) and indicates a slower PWC. This is confirmed by the westerly surface wind anomalies between 150° and 170°W (0.5 ms^−1^) that extend throughout the troposphere (0.7 ms^−1^ at 800 hPa) in the western Pacific (Fig. 2*C*). SOLAR reproduces the pattern of observed zonal wind anomalies over the equatorial Pacific but the model simulates broader easterly anomalies over the Indian Ocean (30°–90°E), associated with overall broader simulated SLP and SST patterns. This is related to a common model bias in simulating tropical Pacific feedbacks, which also affects the SLP, precipitation, and SST responses to ENSO variability on interannual timescales (*SI Appendix*, Fig. S7) and is present in many other coupled atmosphere–ocean models (22). The spread of wind anomalies among individual ensemble members in SOLAR is low and all consistently show a weakened PWC with reduced easterlies in the western Pacific and a dipole of negative/positive anomalies in the upper troposphere (*SI Appendix*, Fig. S8). MSSA filtering of the ensemble mean SOLAR zonal winds at 800 hPa over the western and central Pacific identifies a quasiperiodic (∼10.3-y) fluctuation in phase with the specified TSI variability (∼10.5-y period) over 100 model years with a correlation coefficient 0.6 at lag +1 y (Fig. 2*F*). An eastward shift of the upward branch of the PWC in SOLAR brings more rainfall toward the central Pacific (Fig. 1*H*), with an amplitude and pattern comparable to the observations.

An eastward shift of the PWC following Smax has also been simulated in previous studies (17, 18), but as an additional measure to validate our model responses, we identify a similar pattern of westerly wind anomalies at Smax in the Community Earth System Model (CESM) Large Ensemble Project simulations that include all historical forcings (*SI Appendix*, Fig. S9). In summary, the SOLAR simulations reject the null hypothesis that the PWC response is due to internal decadal variability and instead provide supporting evidence that the observed weakening of the PWC is a genuine response to the increased TSI at solar maximum.

## Weakening of the PWC by a Muted Hydrology

Previous analysis of CMIP5 historical simulations indicates that a starting point in understanding the mechanisms whereby the SC variability influences the PWC is found in the muted hydrological cycle mechanism (18). As direct observations of precipitation suffer from limited spatiotemporal coverage and homogenization issues (23), we turn to the reanalyses to examine global signals at lag +1 y in precipitation and water vapor content (WVC). Reanalyses generally indicate an atmospheric moistening at Smax with increases of 0.5% (*P* < 0.25), 0.7% (*P* < 0.05), and 0.4% (*P* < 0.4) for 20CR, ERA20, and NCEP, respectively (Fig. 3). Over the ocean only (land grid points masked out) the signals are 0.6% (*P* < 0.05), 0.74% (*P* < 0.05), and 0.57% (*P* < 0.15). This translates to a global-mean sensitivity of 6.3%/K, 7.7%/K, and 4.2%/K, under global surface warming (per 1 W/m^2^) of about 0.07, 0.09, and 0.1 K (Fig. 3). While the WVC is largely controlled by the C-C relation, global precipitation changes are constrained by atmospheric energetics and generally increase at a reduced rate in response to a warmer surface (24). The SC imprint on global precipitation is more uncertain than the WVC and reanalyses show insignificant positive anomalies at lag +1 (0.06%, 0.1%, and 0.32% for 20CR, ERA20, and NCEP, respectively, generally *P* < 0.6). However, the sensitivity of global precipitation changes to surface warming (0.94%/K, 1.15%/K, and 3.17%/K, for 20CR, ERA20, and NCEP, respectively) lies within the spread of responses in the AMIP individual runs (range −0.6–4.2%/K at lag +1 y), which show an ensemble mean increase of 1.92%/K. As expected, percentage increases of global WVC are larger than the corresponding precipitation signals in all AMIP runs, with an ensemble mean WVC sensitivity of 8.84%/K (ensemble range 6.1–11.6%/K). Comparable changes in global hydrology are also found in SOLAR at lag +1. The WVC increases significantly by 0.72% (*P* < 0.01 and range 0.4–1%), while global precipitation increases by merely 0.12% (*P* < 0.22 and range 0.07–0.2%), implying an ensemble mean sensitivity to the SC of 9.6%/K for WVC and 1.6%/K for precipitation.

Reanalyses and model results provide evidence for a stronger sensitivity of the global WVC increase versus global precipitation in response to the SC, which is consistent with SC signatures in global hydrology identified in the CMIP5 models and other attribution studies (18, 24). According to theory and sensitivity simulations of GHG forcing (2, 25), the dominant driver of global hydrological changes is the spatially uniform SST response while the SST pattern (longitudinal distribution of SST anomalies) is of secondary importance. To investigate whether this is also the case for solar forcing, we analyze an AMIP-ZM ensemble, which is identical to AMIP but forced only by zonally averaged SST anomalies from 1950 to 2013 superimposed on the SST climatology (*Materials and Methods*). In this way, the longitudinal pattern of SST response to SC forcing is removed and surface temperature anomalies over the Indian and Pacific Oceans are characterized by a zonally uniform warming, which barely exceeds 0.1 K (Fig. 1*L*). Analysis of this ensemble confirms that the global-mean WVC and precipitation increase at almost the same rates as in the AMIP (with differences arising mainly over land; Fig. 3). The muted response of the global hydrology weakens convection and tropical circulation as indicated by the westerly anomalies (up to 300 hPa) in the Far West Pacific (Fig. 2*D*). Westerly wind anomalies in AMIP-ZM are considerably weaker (<0.2 m/s, *P* < 0.4 at 800 hPA) than AMIP and show a wider ensemble spread because of the weaker magnitude of the specified SST gradients (*SI Appendix*, Fig. S10). For this reason, it is not surprising that AMIP-ZM shows insignificant ΔSLP anomalies (Fig. 1*J* and *SI Appendix*, Figs. S2 and S5). However, we detect a significant (*P* < 0.1) increase in precipitation which, like the circulation anomalies, is confined to the West Pacific.

Comparison of the AMIP versus AMIP-ZM simulations suggests that while the general response pattern may be determined by the zonal-mean warming, the amplitude of the surface precipitation and wind response as well as the extent of the eastward shift of the PWC, are probably associated with the longitudinal SST response pattern. It is therefore of interest to explore the cause of this SST warming pattern and how it further weakens the PWC.

## Amplification by the Bjerknes Feedback

It is not well understood why the Pacific warms preferentially in response to increased TSI (Fig. 1*C*). Previous studies have noted an ENSO-like pattern and suggested a dynamically excited mechanism involving a damped-resonant ENSO response triggered by direct TSI surface heating (9). However, the weak westerly anomalies found in AMIP-ZM suggest an alternative mechanism and here we propose that a Bjerknes feedback further weakens the PWC at Smax.

We investigate this hypothesis in a conceptual framework using a low-order atmosphere–ocean model of the Pacific that has been extensively used to study ENSO dynamics (26). The model solves for SST and thermocline depth anomalies in two boxes, representing the east and west equatorial Pacific, by taking into account the Bjerknes feedback that drives wind stress anomalies, which in turn cause equatorial upwelling in the eastern Pacific and zonal advection (*SI Appendix*). Effects of the SC are introduced as an 11-y sinusoidal modulation of the wind stress of 1% amplitude, roughly corresponding to the 10% reduction in surface easterlies simulated in AMIP-ZM. The model shows that the imposed westerly anomaly reduces the thermocline tilt between the West and East Pacific at Smax, resulting in eastward advection of anomalously warm water and positive SST anomalies in the East Pacific (*SI Appendix*, Fig. S11). The west–east SST gradient is thus weakened and hence the Bjerknes feedback reinforces the imposed westerly anomalies. The transition from Smax to Smin weakens and eventually reverses the wind anomalies to easterlies at Smin.

**Fig. 4.**
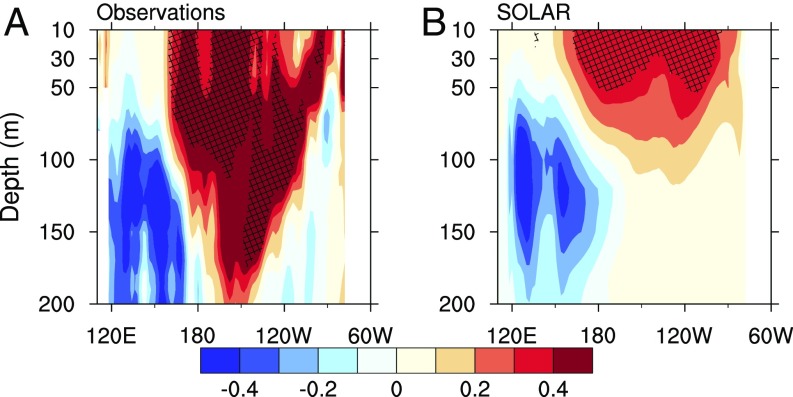
SC regression coefficients of ocean temperature in the equatorial Pacific for (*A*) EN4.2.0 (1950–2013) and (*B*) SOLAR. Hatched areas indicate chance probability *P* < 0.1. Units in K per 1-W/m^2^ increase in TSI. Signals refer to +1-y time lag.

Results of this low-order ENSO model can only frame a theoretical background for understanding the observed PWC weakening given that the simulated response amplitude depends on the assumed coupling parameters. Nevertheless, the low-order model predicts a weakening of the equatorial Pacific thermocline tilt at Smax, and thus a lifted thermocline in the western Pacific that brings cold water to the subsurface. We find further evidence that the two-box model can be considered as a credible analog of the observed SC response by examining equatorial subsurface ocean temperature responses in the EN4 objective analysis for the period 1950–2013 (Fig. 4, *Materials and Methods*). MLR identifies negative temperature anomalies, indicating a shallower thermocline in the western Pacific at +1 y lag (−0.5 K per 1 W/m^2^ TSI at 130 m, *P* < 0.3) and significant positive anomalies indicating a deeper thermocline in the eastern Pacific (>1 K, *P* < 0.05; 120°–160°W at 130-m depth). This means a reduced thermocline tilt at Smax as suggested by the low-order model. This observational evidence is supported by the SOLAR ensemble, which simulates cold water in the western Pacific subsurface, but does not show a stronger warming in the subsurface over the central and eastern sector (Fig. 4). This negligible change in the eastern Pacific thermocline likely explains the generally weaker SST warming in the equatorial Pacific at Smax (Fig. 1*I*).

The low-order model thus highlights the potential for a Bjerknes amplification of the weak westerly wind anomalies initially generated by the SC influence on global hydrology. In addition, the relaxation of the trade winds at Smax is likely to weaken evaporative cooling, which further weakens the surface winds. This wind–evaporation–SST feedback (27), not accounted for in the low-order model, could provide a source of additional warming in the West Pacific, as indicated by the pattern of Pacific SST anomalies in the Kaplan dataset and the SOLAR ensemble (Fig. 1 *C* and *I*).

## Summary and Discussion

Studies of the response to centennial increases in TSI have suggested a contrasting response, with reduced rainfall in the Pacific and stronger east–west SST gradients (28). However, this response is not supported by a recent multimodel comparison of the tropical impacts of a step-change doubling of CO_2_ concentration versus a 2% increase in TSI (29). In those simulations, both forcings produced strikingly similar patterns of increased precipitation over the central Pacific and a relaxed east–west SST gradient, despite the fact that TSI predominately affects the surface, whereas GHGs perturb the radiative balance of the troposphere. In fact, the globally averaged precipitation is expected to increase at higher rates for the same surface warming under TSI than GHG forcing (24). Likewise, we find no evidence for divergent patterns in the Pacific response given that the detected patterns of increased rainfall, relaxed SST gradient, positive ΔSLP anomalies, and a weakened PWC in response to SC resemble modeled responses to 2% TSI and doubling CO_2_, despite subtle differences in the timescale and the magnitude of the forcing (2% TSI roughly corresponds to 20× the SC amplitude).

By highlighting the importance of global hydrology in mediating SC responses in the tropical Pacific, our findings add confidence in the modeled predictions for a weakened PWC, driven by similar hydrological changes in response to GHG forcing. We are not suggesting, however, that observed SC signatures can be used as an analog for quantifying future PWC responses to GHGs. This is because we identify ΔSLP changes of about 300 Pa/K (24–28 Pa per 1 W/m^2^ TSI associated with about 0.08 K per 1 W/m^2^) between solar maximum and minimum, which are considerably larger than the observed century-long ΔSLP trends (50–100 Pa/K scaled to the global-mean SST warming) (4, 5). Reasons for reduced sensitivity in the case of GHG forcing are likely to be found in the pattern of SST response in the Pacific, which is ill constrained in observations and/or cancellation from opposing mechanisms that may not be as effective on the 11-y SC timescale. For example, a long-term adjustment of the thermocline to GHG forcing has been found to reduce the effectiveness of the Bjerknes feedback (30), while our analysis, instead, highlights the importance of this feedback in reinforcing wind and SST anomalies and ultimately the PWC reduction. In summary, we propose a bottom-up pathway by which the SC influence in global hydrology induces weak westerly surface wind anomalies which are then amplified by the Bjerknes ocean–atmosphere feedback (and possibly also a wind–evaporation–SST feedback), leading to the further reduction and eastward shift of the PWC, positive SSTs, and more rainfall in the equatorial Pacific. This study suggests that SC forcing is an appreciable source of decadal variability in the Indo-Pacific and further studies are required to assess the potential to improve decadal predictability in this region.

## Materials and Methods

### Observations and Reanalyses.

Gridded annual mean time series of SLP are taken from the HadSLP2r (1900–2013) global historical reconstruction (31); sea surface winds at 10 m over the period 1950–2011 are taken from the WASWind composite (32). Global precipitation is taken from the GPCC v.7 (1950–2013) (33) and the GPCP v.2.3 Combined Precipitation Dataset (1979–2013) (34). We use the Kaplan SST (1950–2013) dataset (35) and ocean subsurface temperatures (1950–2013) are taken from Met Office Hadley Centre EN Version 4.2.0 objective analysis (36). We additionally make use of annual time series of AMIP, SLP, WVC, total precipitation, and zonal winds from three atmospheric reanalyses: (*i*) the NOAA-CIRES 20th century reanalysis (1950–2011, Version 2c), (*ii*) the ECMWF ERA-20C reanalysis (1950–2010), and (*iii*) NCEP/NCAR (1950–2013).

### Model Simulations.

We carry out model simulations with the middle atmosphere version of ECHAM5, as implemented in the Modular Earth Submodel System (MESSy) framework (37), with and without interactive ocean coupling. An ensemble of six runs (AMIP) from 1940 to 2013 (10-y spin-up) with the atmosphere-only configuration of the model (AGCM) forced only at the lower boundary by the monthly mean AMIP2 SST and sea ice concentrations. All AMIP integrations have been carried out with constant solar irradiance and present-day GHGs, while neglecting volcanic aerosol forcing. Individual model runs branch off from a long control model simulation with climatological boundary. A twin AGCM ensemble of six members (AMIP-ZM) forced at the bottom boundary by zonally averaged AMIP2 SSTs is additionally carried out to isolate the zonally symmetric part of the SST contribution. The AMIP-ZM SSTs is constructed by adding to the monthly climatology (1950–2013) deseasonalized zonal mean SST anomalies. A third ensemble (SOLAR) of four realizations with ECHAM5 interactively coupled to the Max Planck Institute ocean model (MPIOM) is simulated to analyze surface responses to the SC variability specified following the ideal approach of repeating the SC22 (September 1986 to August 1996) 10 times (*SI Appendix*, Fig. S12). Total and spectral irradiances for the SC 22 are taken by the Naval Research Laboratory SSI model v1. Ozone is prescribed as a time-independent climatology, and no other external forcing is considered. SOLAR runs have been initiated from a long control coupled atmosphere–ocean simulation with constant forcings. The analysis covers 100 model years following a spin-up period of 25 y (125 y in total). ECHAM5 has a spectral dynamical core with a triangular truncation at wavenumber 42 (T42) and with 47 levels in the vertical up to 0.01 hPa. The MPIOM model is configured in a GR15 grid (∼1.5° horizontal resolution) with 40 depth levels.

### MLR Model.

We regress lagged solar cycle signals out of annual time series with an MLR model that isolates the linear contribution of four forcings (18), namely (*i*) 11-y solar cycle described by TSI variations filtered out of multidecadal changes, (*ii*) GHG warming described by the CO_2_ equivalent concentration of all major GHGs, (*iii*) major volcanic eruptions described by the aerosol optical depth at 550 nm, and (*iv*) interannual effects of the ENSO phenomenon described by a high-pass filtered (using a Lanczos filter with 8-y cutoff) Niño-3.4 index to account for the ENSO preferred timescale. Time lags in SC signals are inferred by lagging the solar predictor backward/forward in time (−5 to 5 y, step 1 y) with respect to the fitted variable. Lag zero describes changes in phase with SC, whereas delayed signals are indicated in positive lags. The SC regression coefficients are scaled to a 1-W m^−2^ increase of TSI that is “typical” for solar cycles in the last 50 y (e.g., solar cycle 22). Residuals are tested for autocorrelation with a Durbin–Watson test postulating zero autocorrelation and when rejected, regression coefficients and SEs are modified following the Cochrane–Orcutt iterative method. The null hypothesis of zero regression coefficients is rejected with *P* value < 0.1 using a two-tailed *t* test with degrees of freedom *N*-K-1, where *N* is the length of time series reduced by the K number of regressors plus the intercept. The *P* values have been adjusted for the false detection rate following the Benjamini–Hochberg procedure. Finally, the Ljung–Box test identifies insignificant correlations at higher lags in the areas of interest.

## Supplementary Material

Supplementary File
