# Supplementary material for: Slowdown of the Walker circulation at solar cycle maximum
Source: Proc Natl Acad Sci U S A. 2019 Mar 29;116(15):7186–91. doi: 10.1073/pnas.1815060116 (PMC6462076; doi:10.1073/pnas.1815060116)
Supplement: Supplementary File [file pnas.1815060116.sapp.pdf]

SI Appendix

Title: **Slowdown of the Walker circulation at Solar Cycle Maximum**

Authors: Stergios Misios, Lesley J. Gray, Mads F. Knudsen, Christoffer Karoff , Hauke Schmidt and Joanna D. Haigh

Data sources

HadSLP2r**:** https://www.esrl.noaa.gov/psd/data/gridded/data.hadslp2.html

WASWind: http://www.dpac.dpri.kyoto-u.ac.jp/tokinaga/waswind.html

GPCC Precipitation: http://www.esrl.noaa.gov/psd/data/gridded/data.gpcc.html

GPCP v.2.3 Precipitation: http://www.esrl.noaa.gov/psd/data/gridded/data.gpcp.html

Kaplan SST: http://www.esrl.noaa.gov/psd/data/gridded/data.kaplan_sst.html

Met Office Hadley Centre EN ver. 4.2.0: https://www.metoffice.gov.uk/hadobs/en4/download-en4-2-0.html

AMIP2 SST and SIC used in the AMIP and AMIP-ZM ensembles:

https://pcmdi.llnl.gov/mips/amip/amip2/. It must be noted that the AMIP2 SST dataset blends observations which are not identical to those used to compile the Kaplan SST record or the boundary forcing used in reanalyses and hence some small differences in the SC signals are expected.

CESM Large Ensemble

www.pnas.org/cgi/doi/10.1073/pnas.1815060116

Complementary to the SOLAR ensemble, we also analyze SC signals in the CESM Large Ensemble Project, which includes a 42-member ensemble of fully-coupled CESM1 simulations for the period 1920-2005 (http://www.cesm.ucar.edu/projects/community-projects/LENS/data-sets.html). Each member is subject to the same radiative forcing scenario (including among others, 11yr SC, Volcanoes, and GHGs), but begins from a slightly different initial atmospheric state. Our analysis focuses over the 1950-2005 period, common with reanalyses and AMIP simulations. The ensemble mean response (lag +1 year) in CESM-Large Ensemble is characterized by a pattern of westerly wind anomalies of 0.4 ms^{-1} at 800 hPa 150°W-170°W and a dipole of negative /positive anomalies at 200 hPa, which, as with SOLAR, suggests a reduction of the PWC (Fig. S9).

Simple ENSO model

The low-order ENSO model is a simple two-box model that calculates the temperature anomalies in the western and eastern Pacific and thermocline depth anomalies in the western Pacific determined by the recharge/discharge oscillator. The model considers the amplification mechanism of the zonal wind-SST interaction as well as the out-of-phase, negative feedback of the heat content discharge/recharge between equatorial and off-equatorial latitudes. Equations and terminology are given in (1). In our experiments the parameter that measures zonal advection is set to 0.076 so as the model simulates ENSO events of 2-3 years period as a stable mode triggered by white noise of standard deviation 0.1. The model's parameters take values: a=1/180 $days^{-1}$, T_r=29.5 C^{-1}, μ=0.0026 $C^{-1}day^{-1}$, L=15000000 m, h_m=50 m, H=100 m, z_0=75 m, h*=62 m, r=1/400 $days^{-1}$, κ=22 m/C. For the periodically forced experiments, we introduce westerly wind stress anomalies of amplitude 1% between Smax and Smin (square of 10%

reduction surface easterlies 0.2 ms^{-1} simulated in AMIP-ZM) as a sinusoid of 132 months period. With a higher zonal advection parameter (e.g. 0.084), a self-sustained ENSO mode is obtained. In such configuration however, the SC forced runs show a phase locking of ENSO events to the SC, which is not observed. We carry out an ensemble of four SC forced simulations with different noise history. Because of small ensemble size, to match the SOLAR ensemble size, we filter time series with a Lanczos band-pass filter of 10-12 years cut-off periods. This filtering is redundant in the case of large ensemble size (e.g. 100).

Methods

Observations and reanalysis data have been changed to a 2x2° grid prior to the analysis. The SC regression coefficients are adjusted for autocorrelation of the residuals using the Durbin-Watson and the Cochrane-Orcutt iterative method. The critical value of the Durbin-Watson test for 4 predictors in the case of observations, AMIP and AMIP-ZM is set to 1.52 at p-value 0.05. In the case of SOLAR ensemble with MLR of 1 predictor the critical value is 1.58 at p-value 0.05.

Ensemble mean signals in the model simulations are calculated by first compositing ensemble mean time series and then applying the MLR. To assess the ensemble spread the MLR model is additionally applied separately to each ensemble member. Multi-reanalyses signals are calculated in the same way. In Fig. S2, we first calculate ΔSLP anomalies and then regress out the SC influence. Global mean SC signals in Figure 3 are calculated using globally averaged time series.

Figures

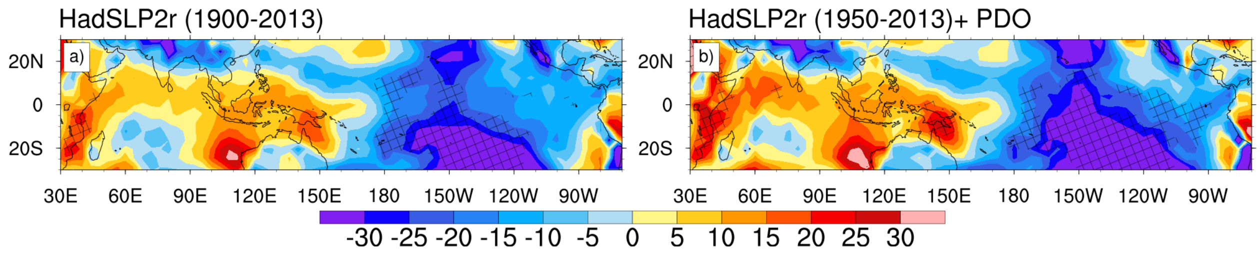

Figure S1 Solar regression coefficients of annual mean tropical SLP for a) HadSLP2r (1900-2013) and b) HadSLP2r (1950-2013) with MLR including additionally the Pacific Decadal Oscillation predictor (http://research.jisao.washington.edu/pdo/PDO.latest.txt). Units in Pa per 1 W/m^2 increase in TSI. All signals refer to +1 year time lag. Chance probability is hashed at p <0.1 .

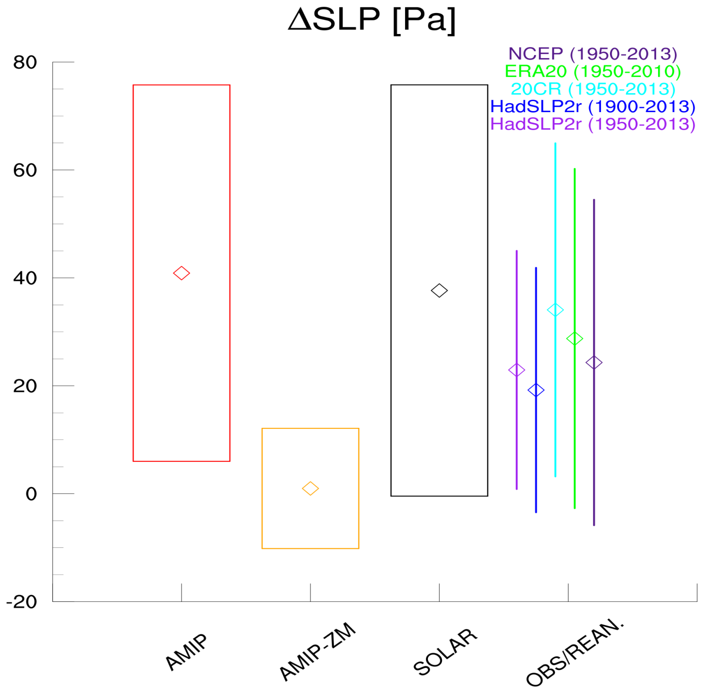

Figure S2 Solar signals in ΔSLP (differences between 160°W-80°W, 5°S-5°N and 80°E-160°E, 5°S-5°N) from HadSLP2r and various reanalysis datasets for years after 1950 and also (in the case of HadSLPr2) from 1900, compared with the corresponding ensemble-mean signals from the AMIP, AMIP-ZM and SOLAR simulations. Mean regression coefficient are denoted by diamonds and 2 standard errors of the mean uncertainties are superimposed. Units in Pa per 1 W/m^2 increase in TSI. All signals refer to +1 year time lag.

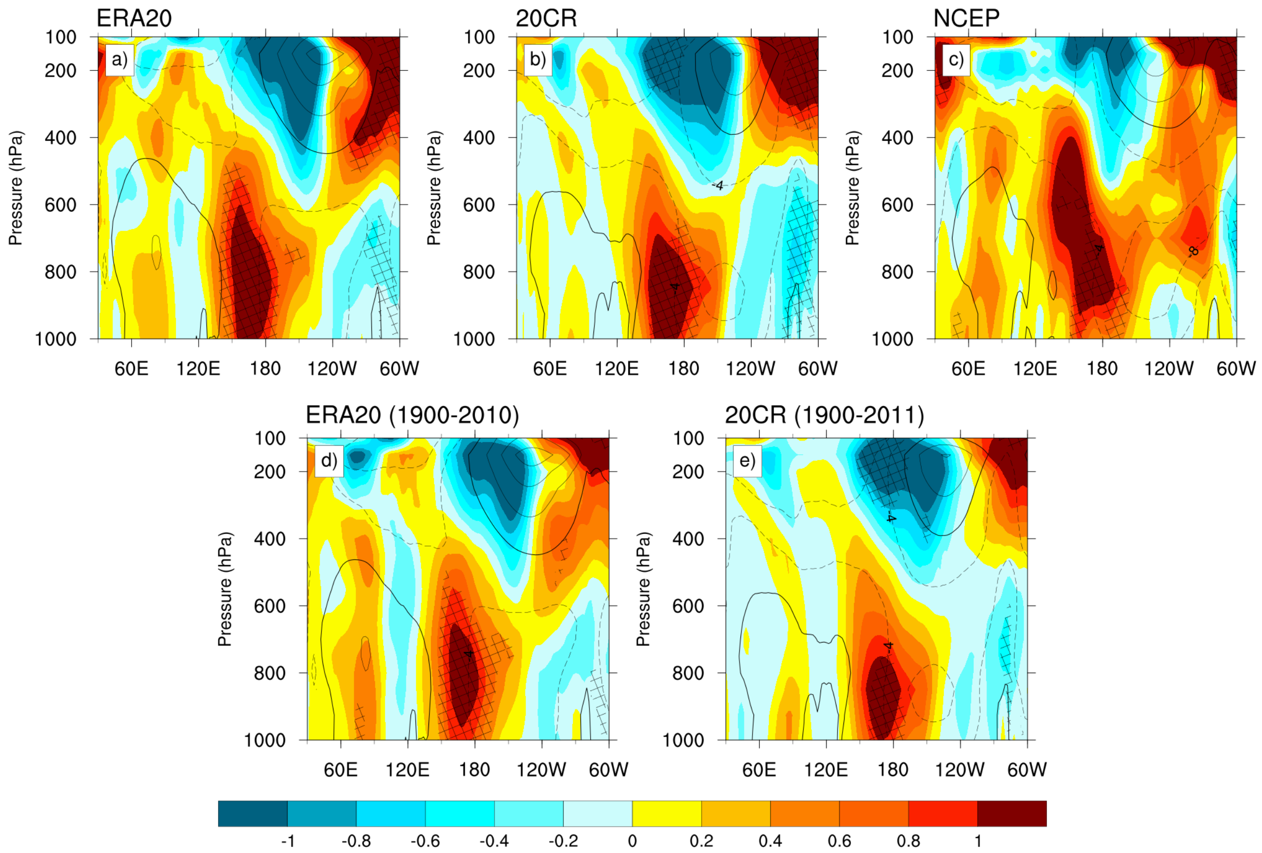

Figure S3 SC regression coefficients of zonal winds averaged over the Equator (5ºS-5ºN) for a) ERA20 (1950-2010), b) 20CR (1950-2011), c) NCEP (1950-2013), d) ERA20 (1900-2010), and e) 20CR (1900-2011). Hatched areas indicate chance probability p <0.1. Contour lines show the zonal wind climatology. Units in ms^{-1} per 1W/m^2 increase in TSI. Signals refer to +1 year time lag.

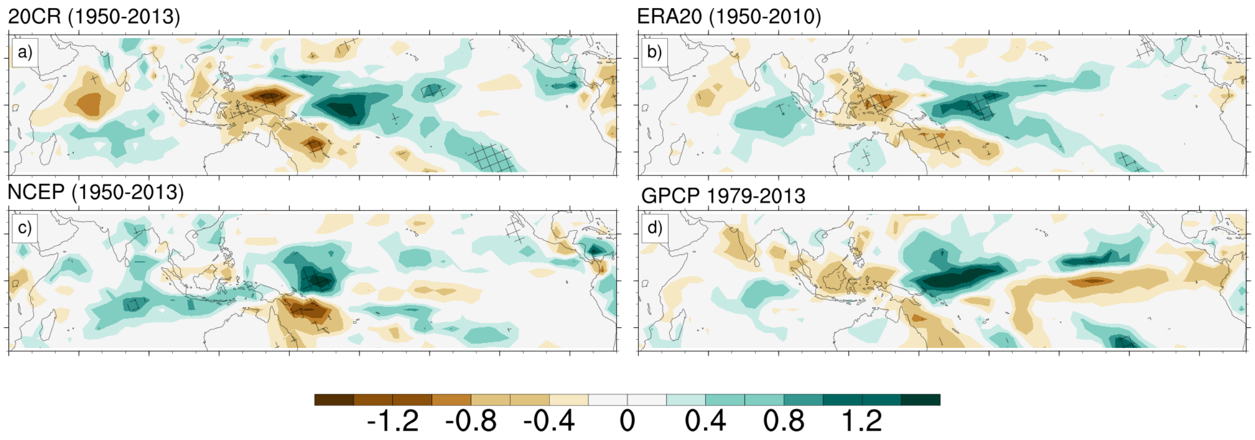

Figure S4 SC regression coefficients of global precipitation for a) 20CR (1950-2013), b) ERA20 (1950-2010), c) NCEP (1950-2013) and d) GPCP v2.3 (1979-2013). Signals refer to +1 year time lag. Units in mm/day per 1W/m^2 increase in TSI. Hatched areas indicate chance probability p <0.1.

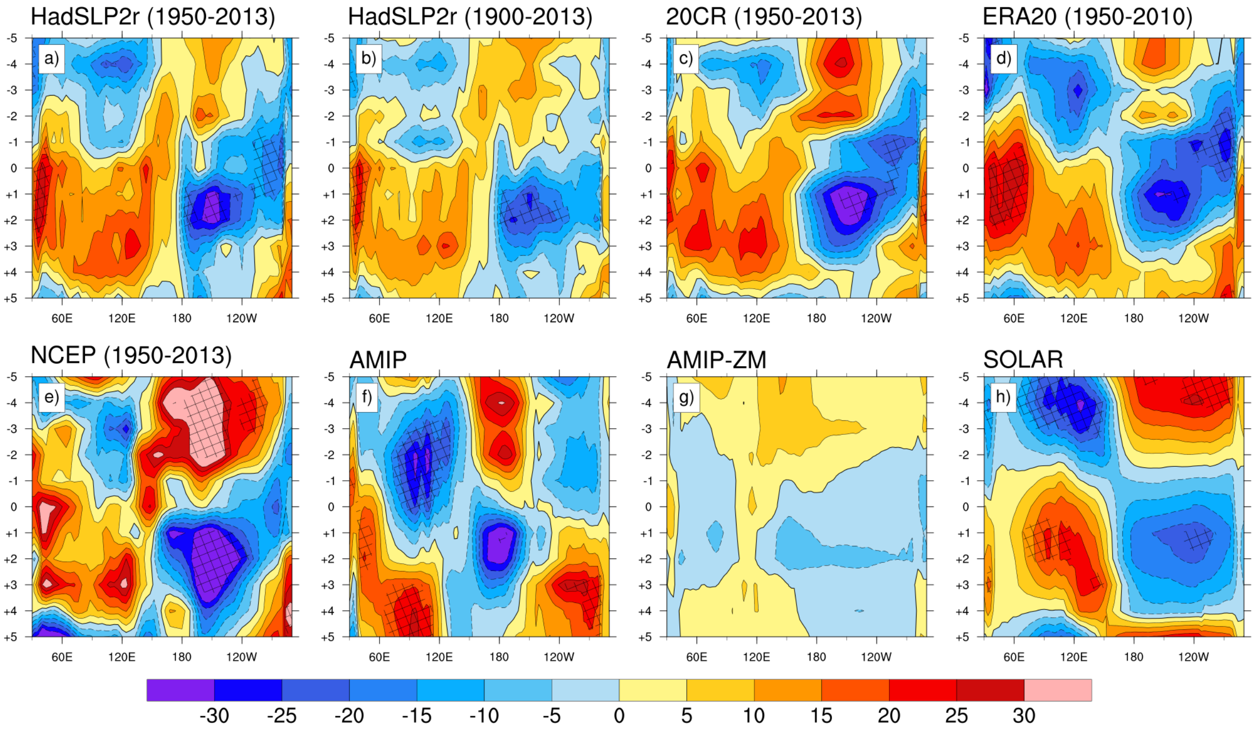

Figure S5 Hovmøller diagrams of lagged SC regression coefficients of annual mean SLP averaged over the equator (5°S-5°N) for a) HadSLP2r (1950-2013), b) HadSLP2r (1900-2013), c) 20CR (1950-2011), d) ERA20 (1950-2010), e) NCEP (1950-2013), f) AMIP, g) AMIP-ZM and h) SOLAR ensembles. Units in Pa per 1W/m^2 increase in TSI. Hatched areas indicate chance probability p <0.1. Positive lags (in years) for a response lagging the SC.

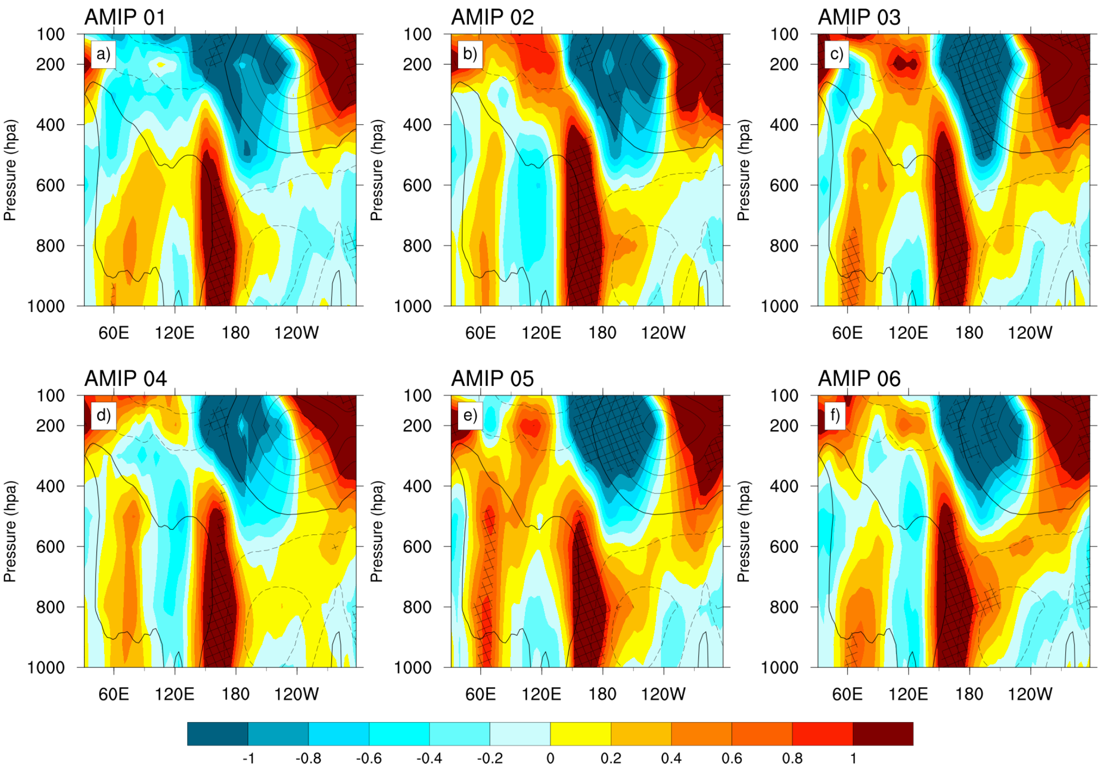

Figure S6 SC regression coefficients of zonal winds averaged over the Equator (5ºS-5ºN) for the six members of the AMIP ensemble. Hatched areas indicate chance probability p <0.1. Contour lines show the zonal wind climatology. Units in ms^{-1} per 1W/m^2 increase in TSI . Signals refer to +1 year time lag.

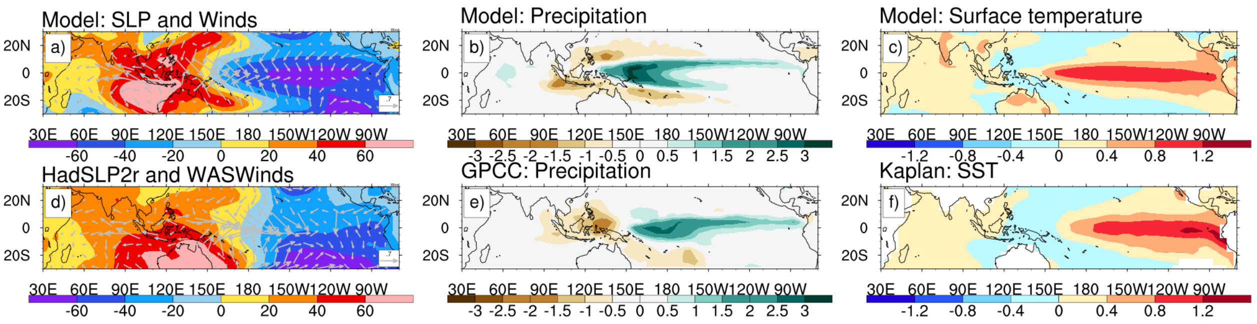

Figure S7 Comparison of patterns in SLP, surface winds, precipitation and surface temperature in response to ENSO in our model (a-c) and observations (d-f). Units Pa, m/s, mm/day, and K in response to 1 K increase in Niño3.4 index.

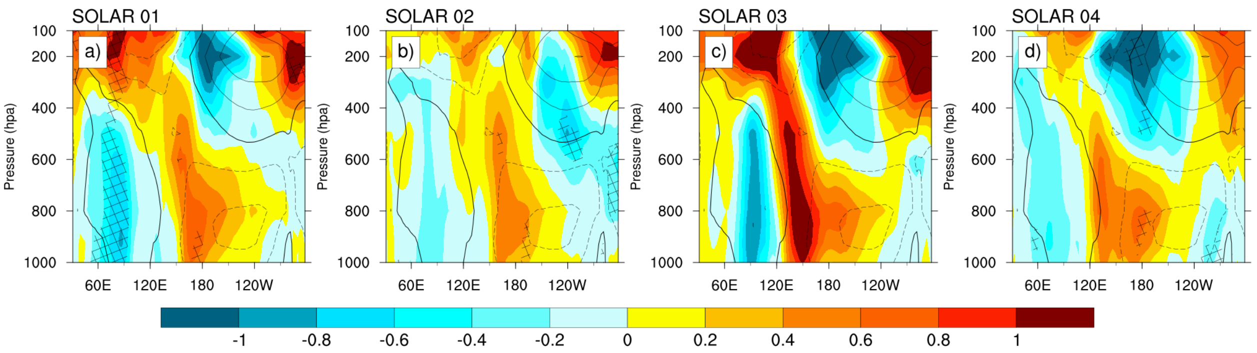

Figure S8 SC regression coefficients of zonal winds averaged over the Equator (5ºS-5ºN) for the four members of the SOLAR ensemble. Hatched areas indicate chance probability p <0.1. Contour lines show the zonal wind climatology. Units in ms^{-1} per $1W/m^2$ increase in TSI. Signals refer to +1 year time lag.

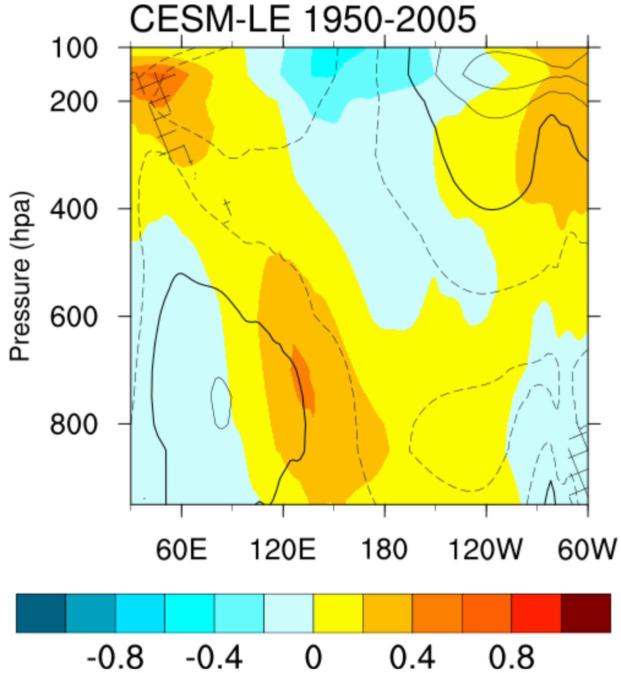

Figure S9 SC regression coefficients of zonal winds averaged over the Equator (5ºS-5ºN) for the CESM-LE simulations over the period 1950-2005. Hatched areas indicate chance probability p <0.1. Contour lines show the zonal wind climatology. Units in ms^{-1} per $1W/m^2$ increase in TSI. Signals refer to +1 year time lag.

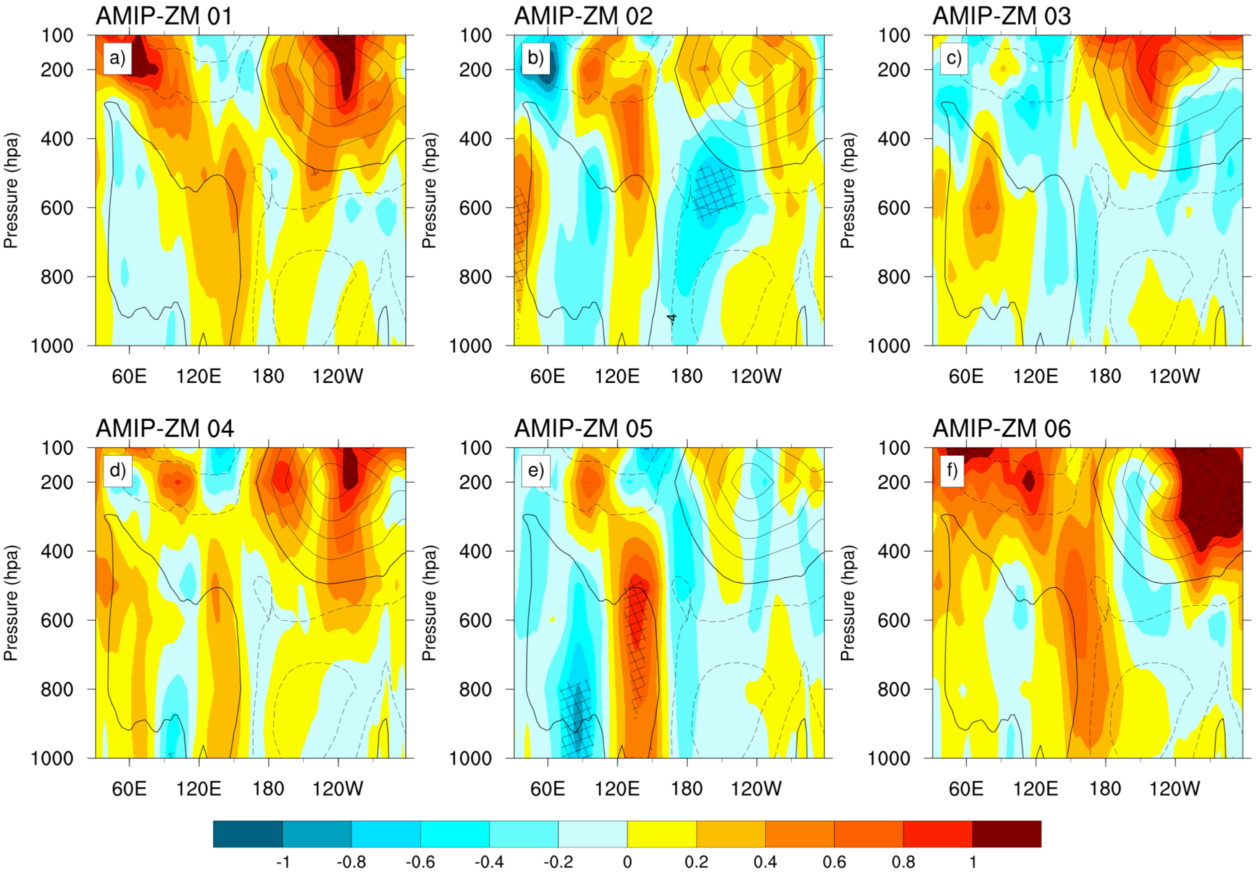

Figure S10 SC regression coefficients of zonal winds averaged over the Equator (5ºS-5ºN) for the ten members of the AMIP-ZM ensemble. Hatched areas indicate chance probability p <0.1. Contour lines show the zonal wind climatology. Units in ms^{-1} per 1W/m^{2} increase in TSI. Signals refer to +1 year time lag.

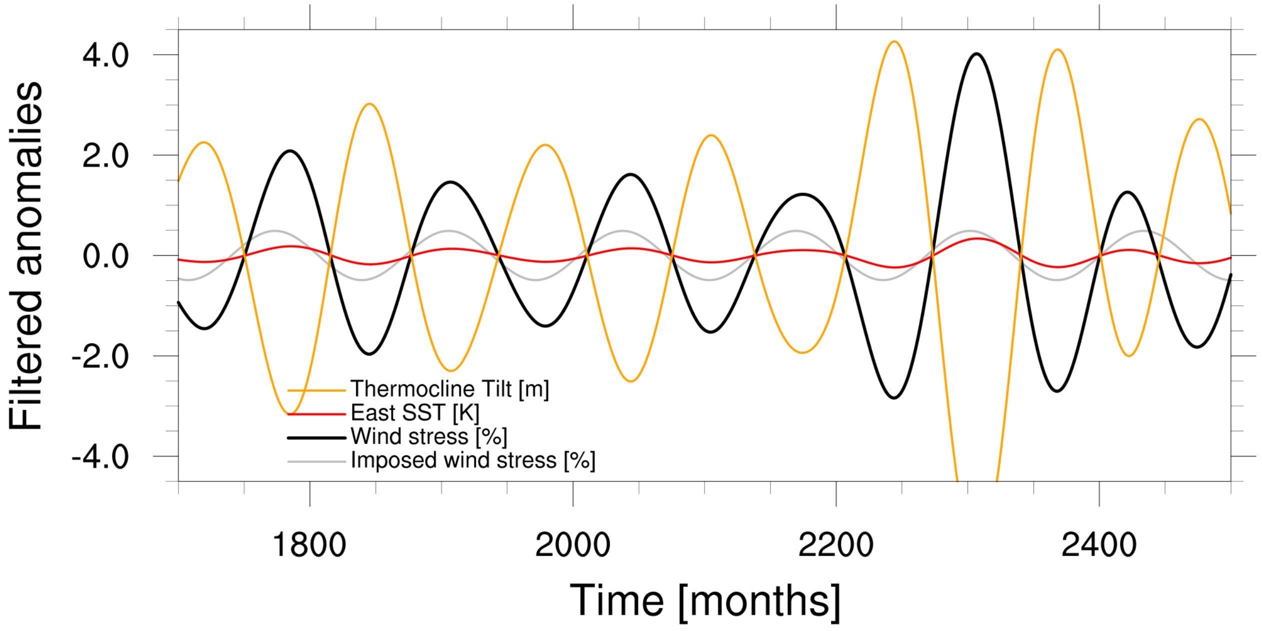

Figure S11 Simulations with a conceptual two-box model of a recharge/discharge oscillator demonstrating the amplification of a sinusoidal (132 months period) wind stress anomaly (grey line) with amplitude taken from the AMIP-ZM. Ensemble mean anomalies of wind stress (%, black), east-box SSTs (K, red) and thermocline tilt between west and east boxes (m, orange). An 10-12-years band-pass Lanczos filter has been applied to the ensemble mean time series. Time in arbitrary months

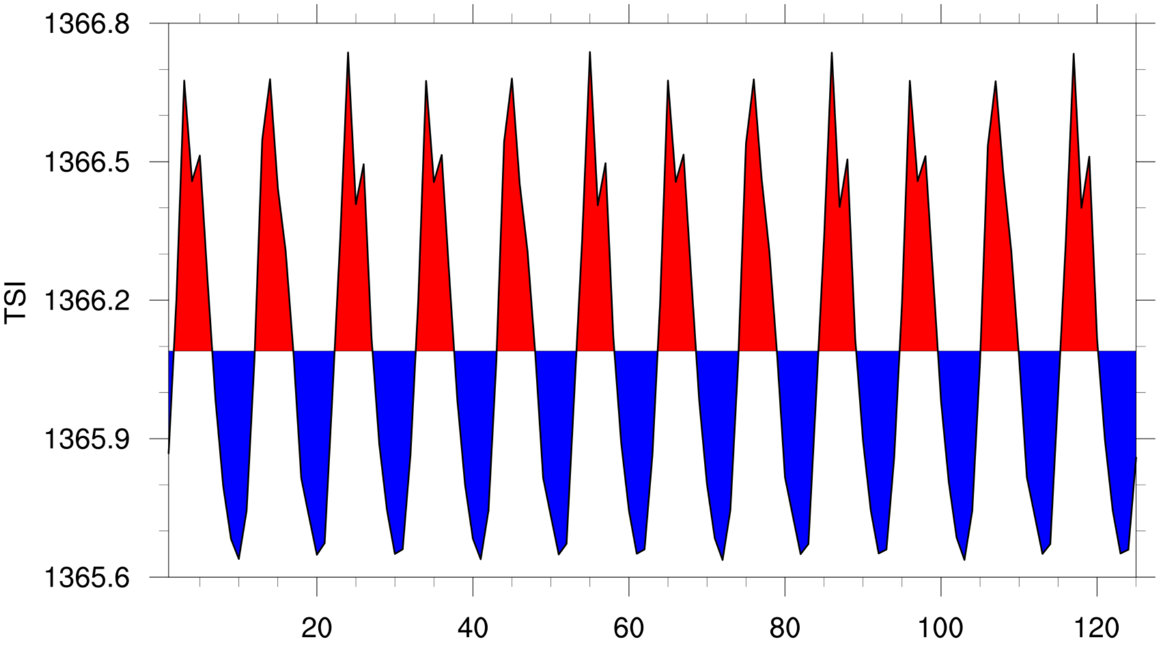

Figure S12 Annual mean TSI specified in the SOLAR simulations constructed by repeating SC 22. The shape of the SC varies over time with a period of 3 SCs because the beginning and end of SC 22 in not synchronized with calendar years. Time in arbitrary model years.

References

1. Timmermann A & Jin FF (2002) A nonlinear mechanism for decadal El Nino amplitude changes. *Geophys. Res. Lett.* 29(1):1003.
